# Blended knowledge sharing model in design professional

**DOI:** 10.1038/s41598-023-43505-z

**Published:** 2023-09-28

**Authors:** Jiaying Hu, Jiyon Lee, Xiuhua Yi

**Affiliations:** 1 College of Art and Design, Nanning Normal University, Guangxi, China; 2 Department of Education Graduate School, Sehan University, Mokpo, Korea; 3 College of Music, Hubei Normal University, Huangshi, Hubei China

**Keywords:** Psychology, Human behaviour

## Abstract

Grounded in Nonaka and Takeuchi (Long Range Plan 54(4):102070, 2021) Socialization, Externalization, Combination, and Internalization (SECI) model, the present research develops a Blended Knowledge Sharing Activity (BKSA) model tailored for design practitioners, targeting the enhancement of learning outcomes and creativity. The investigation centers around the influence of BKSA on higher education students' learning achievements and creative potential, further delving into their application and performance relative to social media within design-related coursework. Employing a comprehensive methodological approach including sampling, t-tests, and structural equation modeling, questionnaires were disseminated to a cohort of 105 undergraduate students from two sophomore-level design classes. It is worth underscoring that despite the SECI model finding extensive applicability across numerous domains, its implementation within the context of design education remains comparatively underrepresented. This research lacuna served as a catalyst in our endeavor to apply the SECI model within knowledge-sharing activities specific to design majors, in anticipation of uncovering more potent strategies for learning and innovation. Our findings disclose a tangible positive correlation between BKSA and both the learning outcomes and creativity of undergraduate students. Moreover, the instrument we devised and utilized, acting as a robust measurement tool for the SECI model, provided additional validation for the beneficial influence of BKSA on university students' learning achievements and creative capacities. This novel insight not only redresses the underexplored application of the SECI model in design education but also furnishes a fresh theoretical vantage point for the amalgamation of blended learning and knowledge sharing paradigms.

## Introduction

The traditional education model in universities has undergone a transformation into a dynamic educational approach^1^. Applying knowledge sharing activities within this dynamic educational framework to design education can enhance the learning outcomes. However, design majors often exhibit weak foundational skills, lack creativity, and engage in online plagiarism as a habitual practice^2–4^. These issues contribute to students' passive thinking behaviors and hinder their creativity. The advent of the internet and social media as platforms for design communication has presented both unprecedented opportunities and challenges for information sharing, knowledge creation, and collective action^5^. Consequently, social media design has become a major strategy for corporate marketing^6^.

The current design courses predominantly rely on traditional offline teaching methods, which are incongruent with the trend towards information technology and open course development^7^. This traditional teacher-centered classroom setup fosters one-way interaction, leading to decreased student engagement^8^. Given the aforementioned context, knowledge interaction and sharing among students within design majors emerge as crucial issues. While Guo et al.^9^ attempted teaching reforms in design courses by integrating them with new media, some scholars have noted that students still harbor concerns regarding knowledge sharing practices^10,11^. Merely using online platforms does not guarantee enhanced knowledge-sharing activities, and students have not yet experienced the positive effects of adequate feedback due to their limited understanding of knowledge-sharing behaviors and principles^10^. Consequently, undergraduate students' limited knowledge and lack of practice result in insufficient knowledge sharing among colleges, schools, and peer groups.

To address the issue of insufficient sharing within design education, Prefume^12^ proposed a combination of face-to-face teaching and online technology. However, current meeting tools are limited by technology, leading to a lack of interaction and sharing between teachers and students^13–15^. Blended learning activities have shown promise in enhancing student memory, autonomy, and sense of responsibility for learning^16,17^. By leveraging social media, blended learning methods provide an additional advantage to design students^17^. Blended learning combines the strengths of traditional learning approaches with networked learning^18–20^, with teachers playing a guiding, inspiring, and monitoring role in the teaching process.

To address the needs of design students, this paper proposes a SECI model of blended knowledge sharing activities. This model integrates internet resources into the learning process, facilitating student interaction on social media platforms. It enables active communication and sharing among students, fosters the integration of internet-based knowledge resources, and facilitates interaction with networked learners on social media.

## Literature review

**Figure 1:**
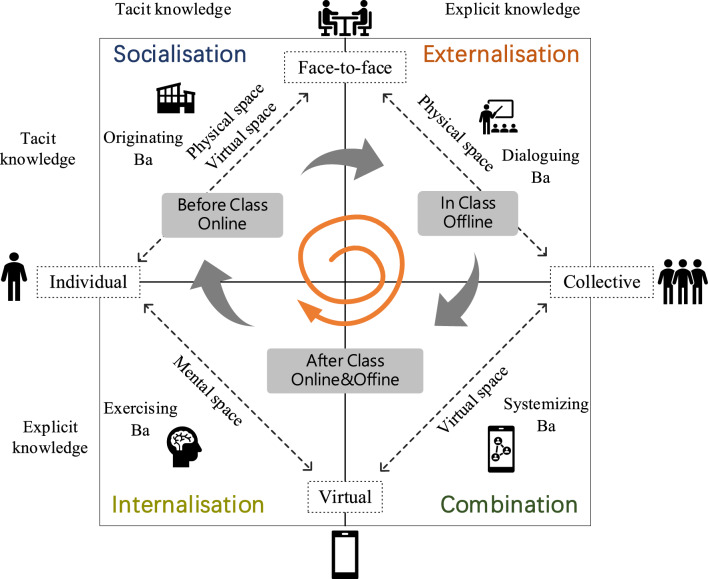
Schematic diagram of the mechanism of blended knowledge sharing.

Since Nonaka^21^ proposed the concept of knowledge sharing, knowledge sharing among individuals, teams, organizations and across organizations has received increasing attention^22^. In the study of knowledge sharing, an important theory involved is knowledge conversion, which is the result of the mutual conversion process between tacit knowledge and explicit knowledge^23–25^. In the design of instructional activities, the concept of knowledge transfer was used in this study. The Socialization Externalization Combination Internalization (SECI) model is combined with blended learning, and the content of blended knowledge sharing activities is designed, see Fig. 1.

### Knowledge conversion theory of SECI model

Knowledge Conversion Theory can be used to explain the interaction between tacit knowledge and explicit knowledge, which was proposed by Nonaka^21^ after an in-depth study of Japanese firms' knowledge innovation experience. The SECI model of Knowledge Conversion Theory is designed in the blended knowledge sharing activity (BKSA), which is an iterative model of organizational learning based on the interaction of tacit knowledge and explicit knowledge. The SECI model, originally proposed by Nonaka^21^, is an important theory to explain knowledge sharing, including Socialization, Externalization, Combination, and Internalization, see Fig. 1.

### Spiral of organizational knowledge creation

In Fig. 1, the first stage is the observation stage, which is in the Socialization mode of the knowledge creation spiral. The socialization model involves tacit knowledge sharing at the interpersonal level, which is defined as the “how to do things” model^26^; The second stage is the recording stage, which is in the Externalization mode in SOKC. Tacit knowledge can be encoded in effective ways for individuals using dialogue and metaphor. For the Externalization model to succeed, distances are inserted between the subject and the object, thereby eliminating the embedding of knowledge^27^. Individuals attempt to codify their tacit knowledge for the team and organizational levels^28^; The third stage is the collation and sharing stage, which is in the Combination mode in the knowledge creation spiral. Explicit knowledge is merged, edited, or processed to form a more systematic explicit knowledge. Creative use of computer communication networks and large databases can facilitate this mode of knowledge transfer. Communication and sharing of information based on ICTs such as virtual communities, intranets, group files, and online databases^29^, can create a higher level of knowledge by using models, best practices, manuals, and information systems^30^; The fourth stage is the practice and practice stage, which is in the Internalization mode in the knowledge creation spiral. Formal knowledge is linked to personal experience and subsequently transferred and used in practical situations^26^. Explicit knowledge is absorbed by individuals, enriching one's tacit knowledge base.

### SECI and BA

Recent research shows that the SECI model has been successfully applied in Knowledge Creation in education^31,32^. Nonaka and Yamaguchi^33^ showed that the combination stage implies that abstract concepts can be turned into concrete action plans. For the combination stage of this study, students converted tacit knowledge into explicit knowledge in the classroom, in cyber Ba, they reorganized their knowledge in the design course. For example, in class, students learned how to repair and restore old photos. Students can edit the learned tacit and explicit knowledge into a new tutorial in the combination stage, such as showing the process of repairing old photos. Sharing knowledge on social networks, students need to reorganize classroom knowledge to form new knowledge sequences and tutorials. SECI also connects an important concept, Ba, defined as a shared environment. Ba originated in Japanese and means "place". Nonaka and Konno^34^ introduced Ba into the knowledge creation model, which can also be interpreted as a place where knowledge is shared^35^, see Fig. 1. Ba can be defined as a spatiotemporal relations, establishing the correlation of space and time, which can unify the physical space, cyberspace, and thinking space^35^.

### Relations between KS, learning outcomes, and creativity

The concepts, characteristics, theories and models of knowledge sharing have been extensively studied^24,36–42^, the results show that knowledge sharing activities are effective in improving students' learning outcomes and creativity. Regarding the relation between the KS and the design majors, design majors need to improve their learning outcomes, which can better adapt to career positions and understand and apply design expertise^43^. Design students need to increase their creativity to avoid uninspired plagiarism^2^.

KS and Learning Outcomes. Regarding the KS and learning outcomes, some scholars have studied the relationship between the two. An experimental study based on the SECI model Wallace^44^ found that individuals can develop tacit knowledge in a shared environment. Sharing experiences fosters empathy and common ground, creating the conditions for effective teacher professional learning teams. Smuts et al.^45^ applied Bloom’s taxonomy to the SECI model and established a new educational knowledge operation model. The results show that experience can transform knowledge and new knowledge into competencies and operational capabilities. The educational knowledge operation model presents an extended spiral relation, the knowledge transformation stage is at the center of the spiral, while the learning stage is at the edge of the spiral.

KS and Creativity. There is a positive relation between the KS and the creativity, with employees' creativity increasing when information is shared or feedback is exchanged among colleagues^46,47^. The creative connection and reorganization of existing knowledge can create new useful knowledge, which also has a certain degree of impact on creativity^48^. Knowledge sharing promotes creativity at the individual level, as KS not only implies the reorganization and efficient transfer of knowledge, skills and information, but also the creation of new knowledge and innovative ideas^49,50^.

In design courses, the development of Bloom's taxonomy corresponds to the SECI model as follows. In design courses, the first level of Bloom's taxonomy remember, corresponds to the internalization stage of the SECI model, which refers to seeking and recalling design design knowledge. The second level of understand in Bloom's taxonomy corresponds to the socialization stage of the SECI model, which refers to the transformation of shared information and experience through design design knowledge into tacit knowledge among individuals. The third level of Bloom's taxonomy, apply, corresponds to the externalization stage of the SECI model, which refers to the use of conceptualization and extraction to facilitate the expression of design design tacit knowledge as explicit knowledge through teacher instruction and peer collaboration. The fourth level of Bloom's taxonomy, analysis, corresponds to the combination stage of the SECI model, which involves sorting, adding, combining, and classifying the collected design design knowledge information, editing it into a new design design knowledge learning tutorial, and sharing it in the network. The knowledge sharing model is a spiral form, so the fifth level of Bloom's taxonomy, evaluate, corresponds to the internalization stage of the SECI model, where students evaluate, debate, and select design design knowledge, form their own opinions based on network feedback, etc. The sixth level of Bloom's taxonomy, create, corresponds to the socialization stage of the SECI model, through the process of creating students' own tacit knowledge of design design, which ends the knowledge operation cycle as understanding deepens and leads to a new hybrid knowledge sharing mode of operation. In each stage of the SECI model, the content of creativity in design professional courses: Divergent thinking includes a love of questions, imagination, curiosity, a wide range of interests, open-mindedness, and a spirit of exploration; the ability to apply talents includes having intelligence, a rich knowledge base, and high flexibility; Personality traits include self-confidence, acceptance of challenges, full of personality traits include being confident, accepting challenges, full of personality, and being different.

### Knowledge sharing activities (KSA)

In this paper, the design of KSA is based on the SECI model, due to the SECI model Nonaka and Takeuchi^51,52^ having been widely adopted in the education field. The knowledge transfer method embodies the knowledge transfer and transfer level learning process, enabling students to use knowledge consciously^53^. The SECI model is not only used in enterprises and organizations, but also used by teachers in teaching activities. The roles of managers, employees, and managers used in the SECI model play the roles of teachers, students, and group leaders in education^24,25^. The field of knowledge sharing is denoted by "Ba", and Naeve et al.,^54^ suggest that Ba exists in many levels, and these levels are connected to form a larger "Ba". Naeve et al.^54^ founded that learning behaviors were present in each of SECI's four knowledge transfer stages.

From the Socialization, Externalization, Combination, and Internalization, scholars have proposed fifteen common strategies and activities^25,28,35,55^. For socialization, tacit knowledge accumulation and transfer can be formed outside the company and in content collection information,For externalization, communication dialogues concretize knowledge so that it can be imparted; For combination, integrate and process knowledge, and then disseminate it; For internalization, participants learn by doing and gain more experience. Both tacit and explicit knowledge have been further developed through simulations and experiments.

According to the principles of teaching activities, this paper selects eleven KSA strategies, as shown in Table 1. Some strategies were not selected as KSA strategies, such as "Information Gathering (Outside the Company)", "Information Gathering (Intracompany)", and "Technical Engagement". It was not used because Nonaka and other researchers worked in the context of businesses and organizations. In addition, the "synthesis and processing" strategy in the KSA strategy is an iterative process, which is not adopted in this paper. Based on the above analysis, the strategies in Table 1 are often mentioned by scholars and can be regarded as the core strategies of KSA activities.

**Table 1:** Strategies and activity content for KSA.

Dimension	Strategy	Activity content
Socialization	① Tacit knowledge accumulation	- Gathering information - Sharing experiences - Observation - Imitating
	② Tacit knowledge transfer	- Share resources - Learn from the masters
Externalization	③ Transmission	- Demonstrations
	④ Communication dialogue	- Facilitating dialogue - Use metaphor - Reporting ideas, opinions and suggestions - Guidance
	⑤ Knowledge concretization	- Taking notes - Creating hypotheses and concepts
Combination	⑥ Access and integration	- Planning, strategy, operations - Collating information - Gathering information - Exploring the Internet - Collecting case - Creating database - Summarize materials
	⑦ Dissemination	- Assignment-based discussion - Presenting innovative ideas - Disseminating new concepts
Internalization	⑧ Access to experience	- More participation activities - Finding and sharing - Communicating and sharing
	⑨ Virtual and experimental	- Participating in experiments - Facilitating challenges - Group experiments
	⑩ Learning by doing	- Learning by doing - Applying theory
	⑪ Development	- Launching socialization - Sharing results

However, the impact of the SECI model process on learning outcomes and creativity has not been considered. For knowledge sharing in college courses, existing studies^56,57^ have shown that knowledge-sharing behavior can significantly improve students' performance and learning ability. Further, on the basis of the existing SECI model^35,58^, this paper combines blended learning and sharing Ba, and designs a learning activity based on SECI model, which is the blended knowledge sharing activity of this paper. Through scales and interviews, the effects of BKSA and its impact on students' learning outcomes and creativity were studied.

The value of knowledge sharing (KS) among individuals, teams, organizations, and on an inter-organizational scale has been comprehensively probed in numerous preceding investigations^22^. Nevertheless, this study leans on the insightful examination performed by Yilmaz and Karaoglan Yilmaz^19^, which focused on the influence of role assignment in online discussions vis-à-vis the perception of transactional distance and the behavior of knowledge sharing. Within the purview of a quasi-experimental study design spanning a duration of 10 weeks and involving 111 undergraduate students, they discerned that the structuring of online dialogues, facilitated by the assignment of specific roles to students (such as initiator, moderator, debater, resource seeker, and summarizer, among others), can significantly attenuate students' perceived transactional distance and enhance their knowledge-sharing behavior. This research illumines novel and substantial insights into the challenges and prospects associated with online discussions, thereby offering pragmatic strategies for educators and students alike to design and engage in high-quality online dialogues. The findings underpin our understanding of the dynamics of knowledge sharing in digital environments, contributing significantly to the refinement of online pedagogical approaches.

Furthermore,^59^ embarked on a meticulous investigation into the interplay among university students' knowledge sharing behaviors (KSB), academic self-efficacy (ASE), and sense of community (SoC) within Facebook-based learning communities. Employing a cogent research design, data were amassed from 316 university students and subjected to rigorous analysis via structural equation modeling (SEM).The findings of their research demonstrated that students' ASE and SoC exerted a positive influence on their KSB. They elucidated the motivations and determinants of knowledge sharing in online communities within the theoretical constructs of the social constructivism paradigm and social cognitive learning theory. Their empirical investigation, using Facebook as a conduit for virtual learning communities, illustrated how such social media tools can be adroitly utilized to amplify learning interactions and collaborations both within and beyond the classroom context. Such revelations serve as a pivotal contribution to our understanding of how digital tools can be harnessed to foster a knowledge sharing environment conducive to the enhancement of learning outcomes.

Simultaneously, some scholars have harnessed the flipped classroom (FC) pedagogical model as a strategy to augment student engagement and improve learning outcomes^20^. These investigations have demonstrated that student participation in FC, bolstered by a Facebook-supported virtual learning community (VLC), is contingent upon their perception of sociability, sense of community, and course satisfaction. These variables emerged as substantial predictors of students' behavioral, cognitive, and emotional engagement within the context of FC. Such research offers a comprehensive theoretical framework that positions perceived sociability, sense of community, and course satisfaction as antecedent variables impacting student engagement. They have confirmed the associations among these variables through a meticulous stepwise multiple linear regression analysis. Furthermore, these studies extend compelling suggestions for future research directions, such as the utilization of other social networking tools, exploration of other potential influencing factors, and the expansion of sample sizes and diversity, among others. This continuous research endeavor opens up new perspectives in our understanding of the intricacies of student engagement in contemporary pedagogical models.

The research led by Karaoglan-Yilmaz et al.^18^ offers a novel perspective on understanding blended learning environments. This scholarly work meticulously dissects the advantages and challenges intrinsic to blended learning and furnishes strategies for the efficacious design and implementation of a blended learning environment. Concurrently, the study illuminates the influence of metacognitive awareness, reflective thinking, problem-solving, and inquiry community on students' self-efficacy, and elucidates the relationships among these variables. This exploration is instrumental in comprehending how students bolster their self-efficacy, technical aptitude, and advanced cognitive skills within a blended learning environment, offering significant theoretical guidance. Further, the investigation conducted by Ustun et al.^60^ devised a scale based on the UTAUT model for educational VR acceptance, measuring students' acceptance and utilization of VR systems. Through three distinct phases of data collection and analysis involving 440 undergraduates across various majors and grades, the authors validated the reliability and robustness of the scale. The scale, encompassing 18 items distributed across four factors (performance expectations, social influences, effort expectations, and facilitating conditions), accounts for 67.62% of the total variance, in alignment with the UTAUT model. Their seminal work bridges a gap in devising a scale to assess VR acceptance specifically tailored to the realm of educational virtual reality, and it presents a roadmap to effectively design and implement virtual reality instructional activities. The scale can be deployed to differentiate among student cohorts across various disciplines, grades, or backgrounds, as well as to measure the impact of virtual reality instructional interventions. Moreover, this scale can be integrated with other variables (such as academic performance, satisfaction, cognitive load, etc.) to probe the effects of VR instruction on learning processes and outcomes.

In the contemporary educational milieu, blended learning and knowledge sharing are emerging as increasingly pivotal. These avant-garde educational modalities offer unique merits, including enhanced flexibility, broader accessibility, and a wealth of teaching resources. The investigations reviewed herein enable a deeper understanding of how to amplify learning outcomes and creativity amongst design students through knowledge sharing, providing invaluable guidance for the praxis of design education.

Drawing on these recent insights, we formulate two hypotheses. The first postulates that blended knowledge sharing activities exert a positive influence on learning outcomes within design colleges. The second conjecture suggests that blended knowledge sharing activities positively bolster creativity within design institutions. These hypotheses will be subjected to rigorous verification in subsequent studies. As such, further exploration and discourse are requisite to comprehend how to enhance the learning outcomes and creativity of design students through knowledge sharing. Specifically, there is a pressing need to research and devise more precise and efficacious knowledge sharing strategies and activities to optimally boost the learning outcomes and creativity of design students.

## Blended knowledge sharing activities

Combining the concepts of blended learning and “Ba”, a new concept, Blended Knowledge Sharing Activities (BKSA), is proposed in this paper. The BKSA of the SECI model, including the respective “Ba”, the framework and content are designed, where blended includes a mix of learning styles and “Ba”. Learning methods include online learning and offline learning, where online learning refers to the learning behavior of the network. Use online resources to learn and share learning knowledge on social media; Offline learning refers to learning behaviors in real life, face-to-face teaching in the classroom. “Ba” is a place for knowledge sharing, which can also be interpreted as a space–time relation. For design courses, BKSA establishes the spatial and temporal correlations of participants in the course of learning activities, where spaces include physical space, virtual space, and mental space. Physical space includes classrooms, dormitories, libraries, etc.; virtual space includes networking, social media, digital media, etc.; mental space includes thoughts, opinions, and perceptions. In addition, BKSA is not limited by time, in other words, learning sharing activities can be conducted at any time. Therefore, BKSA can be mixed with Ba online and offline.

BKSA includes three stages: before class, during class and after class. There are detailed regulations on the learning activity scene, which provides a reference for the formulation of the activity and the behavior of the participants. In the design course BKSA, the core content of the socialization model is that students observe the experience of others and absorb the tacit knowledge of others as their own tacit knowledge; The content of Externalization includes the telling behavior of teachers and the expressing behavior of students. Use dialogues and metaphors to encode tacit knowledge into identifiable and effective methods; The content of the combination mode integrates and innovates the new knowledge learned and recorded, and shares the new knowledge on social media to get a wider range of responses; Internalization mode means that practical activities enable students to integrate new knowledge into their own mental models, enrich professional skills, and provide a basis for generating new tacit knowledge components. The internalized knowledge of the internalization mode circulates in the knowledge spiral, starting the next transformation process and forming the spiral structure of knowledge creation.

Regarding the content of the activities, from the perspective of the participants in the SECI model, teachers are not only bystanders of KS activities, but also participants. The role of teachers as knowledge collectors and activity facilitators guides students. Students are the main participants in the activities and the main sharers^25,28,35^. The contents of the corresponding BKSA activities are shown below.

The first stage is the before class stage, which is in the socialization mode of KS. Learning and KS behavior take place at the originating “Ba”. After expert interviews, the socialization phase is achieved through an online model, not an offline model. Because teachers and students have to communicate face-to-face before class, there are certain practical difficulties in implementation, and the flexibility of time and place is not as convenient as online communication. Students conduct conference discussions in small groups, learn from the experiences of others, and translate into their own tacit knowledge.

The second stage is in class stage, which is in the externalization mode of KS. The learning and KS behavior take place at the dialoguing “Ba”. In the physical space, teachers use metaphors to teach tacit knowledge to students. Students can also use metaphors and analogies to express their views in class discussions and exercises. At this stage, students record the tacit knowledge shared by teachers and form documents or pictures for learning.

**Figure 2:**
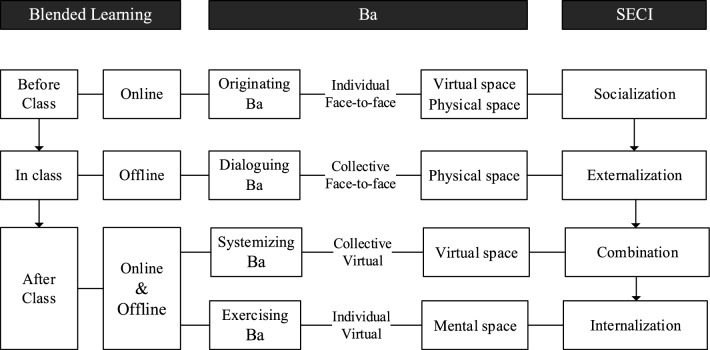
The BKSA framework, including blended learning, Ba, and SECI counterparts.

The third stage is the after-class stage, which includes the combination and internalization mode of KS. Compositions are collective virtual interactions, as shown in Fig. 2. Learning and KS behavior take place at the systemizing “Ba”. In the virtual space, the systemizing “Ba” is the interactive place in the virtual world, not the real space and time. The combination of new explicit knowledge with existing information and knowledge systematically produces explicit knowledge throughout the organization, effectively supporting the combination of explicit knowledge.

## Methods

Adopting a mixed-methods research design, this study amalgamates quantitative and qualitative research methodologies to evaluate the influence of Blended Knowledge Sharing Activities on the learning outcomes and creative capacity of design university students. Mixed-methods research designs strive to achieve a more profound and comprehensive comprehension by capitalizing on the strengths inherent in both quantitative and qualitative research approaches.

### Participants

All the methods were carried out in accordance with institutional guidelines and regulations. Prior to the study, written informed consent was obtained from the research subjects, and all procedures were approved by the Institutional Review Board for Behavioral and Human Movement Sciences at Nanning Normal University. The object of this paper is undergraduate students majoring in design, and two methods of cluster sampling and convenience sampling are used for statistical analysis. Cluster sampling consists of two steps. The first divides the total population into clusters or groups, which are usually geographic locations or regions, such as villages, schools, neighborhoods, and so on. These clusters are randomly selected from experimental samples^61^. Two second-year design classes are selected from University A, with a total of 105 students. These respondents are selected by convenience sampling. After the experiment is completed, ten participants who were willing to answer open-ended questions were selected for interviews. The participants in this study came from two different classes of design majors at University A. School A is an ordinary undergraduate college in Nanning City, Guangxi Zhuang Autonomous Region, China. Its design major has been established for nearly 20 years. The design major recruits about 200 freshmen every year, and the school system lasts for four years. So, the sample is universal. The design major of School A aims to train marketing planners and graphic designers, and requires graduates to have design capabilities in print design design. In the graphic dynamic design, film and television design, and online design industries, the design scheme can be completed independently. Although University A is an ordinary undergraduate university, the major of design is also a major focus of University A. Therefore, it is of universal significance that the design students of University A can be used as research objects. In the implementation of design professional courses, BKSA can improve students' learning effect and creativity in design design learning. Design courses are taught in the second year of university, and the first year of university is generally in the stage of basic courses and public courses. There are generally 4 classes for second-year students in AD, as shown in Table 2. Therefore, the author randomly selected two classes of second-year design majors from University A, with a total of 105 students as the research object. The two classes are divided into two groups, 53 for the experimental group and 52 for the control group. In the experimental group, there are 28 women (52.8%) and 25 men (47.2%). In the control group, there were 29 women (55.8%) and 23 men (44.2%). Most of the students are between the ages of 19 and 20, and they come from urban and rural areas. In the experimental group, 26 students are from urban areas (49.0%) and 27 students are from rural areas (51.0%). The control group consisted of 27 people from urban areas (51.9%) and 25 from rural areas (48.1%). Before the implementation of BKSA, no student had participated in such an experiment. Before the teaching experiment, the demographic variables of the two classes are not significantly different, as shown in Table 2 below. The difference between the experimental group and the control group is almost negligible, which indicates that both the experimental group and the control group can meet the requirements of this experiment.

**Table 2:** Basic information of research participants (N = 105).

Category	Groups	Sub-category	N	Percentage (%)
Gender	Experimental group	Female	28	52.8%
		Male	25	47.2%
	Control group	Female	29	55.8%
		Male	23	44.2%
Birthplace	Experimental group	Urban areas	26	49.0%
		Rural areas	27	51.0%
	Control group	Urban areas	27	51.9%
		Rural areas	25	48.1%

### Procedures

**Figure 3:**
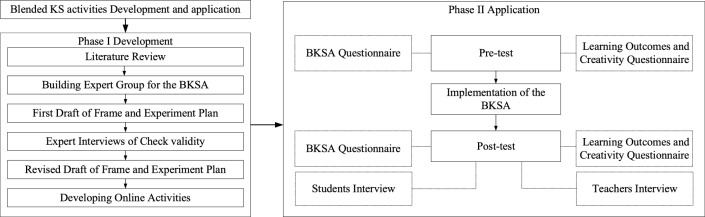
Blended KS activities development and application.

The subjects of this study are undergraduates majoring in design, and cluster sampling and stratified sampling are used for statistical analysis. Cluster sampling consists of two steps, see Fig. 3. First, the total population is divided into clusters or groups. These clusters or groups are usually geographical locations or areas, such as villages, schools, neighborhoods, etc. These clusters are randomly selected from which experimental samples are taken^61^. Two sophomore design classes were selected from University A, with a total of 105 students. These respondents were selected by convenience sampling. After the experiment was completed, ten participants who were willing to answer open-ended questions were selected for interviews. Participants were from two different classes of design majors at University A.

### Instruments

Questionnaires and interviews frequently serve as complementary tools in research, surveys, and educational evaluations. While questionnaires can provide a broad perspective on population patterns, qualitative interview data offer profound insights into participants' attitudes, thoughts, and behaviors^61^. This study employs a combination of these methods to evaluate the efficacy of Blended Knowledge Sharing Activities (BKSA). To examine the validity of BKSA, the study leverages data analysis from both questionnaires and interviews. Firstly, the data from questionnaires on blended knowledge sharing, learning outcomes, and creativity were analyzed using IBM SPSS Statistics. The data underwent a series of tests, including independent samples t-test, paired samples t-test, correlation analysis, and regression analysis. This data analysis allows us to ascertain whether there are differences in learning outcomes and creativity among the two groups of design students post-experiment, and whether blended knowledge sharing influences these factors. The effectiveness of BKSA is then appraised based on these quantitative analysis outcomes. Secondly, the study gathered and analyzed interview data from both experts and students. Interviews were meticulously transcribed and coded for analysis according to sequence category, attribute, and dimension. The focus of these interviews centered around key words or phrases, particularly those frequently employed by the interviewee. When synthesizing these queries, they were conclusively validated through a consensus of experts to ensure the integrity and scientific validity of the data.

#### Blended knowledge sharing Questionnaire

The questionnaire is compiled based on the knowledge Sharing Behavior Questionnaire by Nonaka et al.^62^, Haag and Duan^63^, and Songkram and Chootongchai^64^. The Cronbach's alpha of the questionnaire of Haag and Duan^63^ was 0.878, and the Cronbach's alpha of the questionnaire of Songkram and Chootongchai^64^ was 0.887, which is consistent with the theme of this study. The purpose of the questionnaire is to explore the behavior of students in SECI in the process of knowledge sharing. The questionnaire has high reliability (Cronbach's α = 0.890), as shown in Table 3. The CR values of the combined reliability of the BKSA questionnaire were all greater than 0.7, indicating that the combined reliability of the dimensions is good. The questionnaire has five components, and 16 closed-ended questions. The five components are: socialization, externalization, combination, and internalization. Socialization is the stage in which tacit knowledge is converted into tacit knowledge, and examines students' observation and communication abilities. Externalization is the stage in which tacit knowledge is transformed into explicit knowledge, and it examines students' ability to extract knowledge. Combination is the stage in which explicit knowledge is converted into explicit knowledge, and it examines students' ability to integrate knowledge. Internalization is the stage in which explicit knowledge is transformed into tacit knowledge, which mainly examines students' ability to reflect and comprehend.

**Table 3:** The details of blended knowledge sharing questionnaire.

Dimensions	Items	Title number	Cronbach's α
Socialization	3	5, 6, 7	0.882
Externalization	3	8,9,10	0.857
Combination	4	11,12,13,14	0.865
Internalization	3	15,16,17	0.931
Overall scale	16	-	0.915

#### Learning outcomes Questionnaire

Based on the indicators of learning outcomes in the revised Bloom's taxonomy^65,66^, a questionnaire validity scale of learning effects is designed for design professional courses, as shown in Table 4. This table contains a total of 6 dimensions (each dimension includes 3 items), including remember, understand, apply, analyze, evaluate, and create. The remember represents the degree to which knowledge is remembered. The understand examines the ability to interpret or understand knowledge. The apply means that learners can use information or knowledge in new ways. The analyze is when the learner can distinguish the different parts of things. Evaluate means that learners can justify their position or decision. The create is a test of whether learners can create new products or ideas. Pre-investigation is conducted on the learning effect scale, and the reliability of the questionnaire was analyzed based on IBM SPSS Statistics. The results show that the learning effect questionnaire has high reliability, Cronbach's alpha is 0.912. The reliability CR values of the questionnaires are all greater than 0.7, which means that the reliability of the dimension combination is good. Learning outcomes questionnaires represent different types of cognitive activities that can be used to assess mastery of learning outcomes.

**Table 4:** The validity of learning outcomes questionnaire.

Dimensions	Items	Title number	Cronbach's α
Remember	3	18, 19, 20	0.876
Understand	3	21, 22, 23	0.921
Apply	3	24, 25, 26	0.893
Analyze	3	27, 28, 29	0.852
Evaluate	3	30, 31, 32	0.916
Create	3	33, 34, 35	0.786
Overall scale	18	–	0.912

#### Creativity Questionnaire

The creativity questionnaire (Cronbach's α = 0.867) can usually be divided into divergent thinking, intellectual application ability, and personality traits^67^. Divergent thinking represents an individual's thinking habits, imagination and curiosity, and mainly tests an individual's openness of mind and spirit of exploration. The ability to apply intelligence examines an individual's intelligence and knowledge reserves. Personality traits are measures of an individual's personality and habits. For the design professional course, this paper modifies the specific items of each dimension, designs the items suitable for the design professional course, and obtains the questionnaire validity table of the creativity test, as shown in Table 5. The table has 3 dimensions of measurement and contains 20 items. Through the analysis of IBM SPSS Statistics, the validity of the questionnaire was measured by pre-research on the creativity scale, and the results showed that the creativity questionnaire has a high validity (Cronbach's α = 0.903). The creativity questionnaire represents the innovative ability and potential of individual thinking and behavior.

**Table 5:** The details of creativity Questionnaire.

Dimensions	Items	Title number	Cronbach's α
Divergent thinking	7	39, 40, 41, 42, 43, 44, 45	0.920
Application ability	8	46, 47, 48, 49, 50, 51, 52, 53	0.927
Personality traits	5	54, 55, 56, 57, 58	0.867
Overall scale	20	-	0.903

#### Interviews

This article conducts expert interviews with the purpose of verifying the feasibility of BKSA implementation, including 3 interview questions: i) What do you think of the first draft of the BKSA based on the literature review; Any other suggestions for a blended knowledge -sharing strategy; Do you have any suggestions for the implementation of the BKSA? To obtain the feedback effect of the BKSA experiment, this study interviewed 10 students from the experimental group. They were selected by convenience sampling and all volunteered to participate in semi-structured interviews. All 10 students participated in all courses of the BKSA experiment and the interview questions are shown in Table 6. During these interviews, students stated their thoughts on BKSA and what they felt they gained by participating in the experiment. They also made some recommendations for the future implementation of the BKSA. Later, a detailed discussion will be given in the results.

**Table 6:** Questions for student interviews.

Interviewee	Interview question
Students in experimental group	Q1. How did you start the blended knowledge sharing activity?
	Q2. Can you tell me about your adaptation process to the BKSA course?
	Q3. When you compare BKSA teaching with traditional teaching, do you think this method has advantages? Which method do you like more?
	Q4. What do you think are the limitations of this method compared to traditional education methods?
	Q5. Do you like or dislike BKSA?
	Q6. What are your greatest feelings about this course?
	Q7. What is the learning effect after this course?
	Q8. How has your creativity changed after this course?

We conducted interviews with classroom teachers from the BKSA panel who were involved from the early strategy development stages to the final implementation of the project in response to feedback on the effectiveness of the BKSA. The class teacher raised the following questions: (i) How would you evaluate the BKSA; (ii) What suggestions do you have for BKSA?

## Results and discussion

The objective of this study is to analyses the impact of BKSA on academic performance and creativity. We used questionnaires and interview instruments for quantitative and qualitative testing. The questionnaires analyzed the differences in learning outcomes and creativity between the two groups of students after the experiment. The differences between students in the experimental group before and after the experiment and the effect of blended knowledge sharing activities on learning outcomes and creativity. Interviews were conducted to obtain students' and teachers' evaluations and suggestions about BKSA. The SPSS was used to analyze the basic situation and differences in problem solving skills between the experimental and control groups before and after the measurement, and to discuss the differences in student engagement between the experimental and control groups.

BKSA is a 16-week teaching activity that will begin on September 17, 2021 and end on January 7, 2022. BKSA is a curriculum activity that is used in professional courses for design students to improve their learning outcomes and creativity. The activities allow students to improve not only their learning outcomes, but also their creativity and aid design students to quickly adapt to professional positions. This study course is designed based on the four-stage elements of the SECI model, in which the three stages of blended learning are the main teaching stages. The graphic design design software Photoshop (PS) is used as the teaching content to develop teaching activities using BKSA. The classroom is divided into four steps: socialization-externalization-combination-internalization. Teachers design teaching activities based on the content of learning outcomes. BKSA makes full use of the advantages of KS, applies cooperative learning and knowledge sharing to different parts of the course, and each part is closely connected, and the activity design shows the characteristics and advantages of BKSA activities. This study constructs a basic environment for online activities of the design course BKSA on social media (SM) based on the BKSA structure, content and activities, and its feasibility is verified by experts.

### The effects of BKSA on learning outcomes

Table 7 presents a comparison of the pre-test scores between the experimental group and the control group across the cognitive domains of remembering, understanding, applying, analyzing, evaluating, and creating. The differences between the two groups in these aspects were not statistically significant (*p* > 0.05) during the pre-test, suggesting that the two groups were homogeneous at this stage of the experiment.

**Table 7:** Pre-test and post-test comparison between experimental group and control group for learning outcomes.

Dimension	Group	Test	M	SD	t	p
Remember	Experimental group	pre-test	3.03	0.97	0.10	0.92
		post-test	3.21	0.87		
	Control group	pre-test	3.01	0.97	0.30	0.76
		post-test	3.17	0.72		
Understand	Experimental group	pre-test	3.06	0.96	− 0.01	0.99
		post-test	3.22	1.05		
	Control group	pre-test	3.06	0.97	0.15	0.88
		post-test	3.19	0.76		
Apply	Experimental group	pre-test	3.04	0.96	0.10	0.92
		post-test	3.21	1.05		
	Control group	pre-test	3.02	0.96	0.05	0.96
		post-test	3.20	0.64		
Analyze	Experimental group	pre-test	3.02	0.91	0.11	0.92
		post-test	3.38	0.61		
	Control group	pre-test	3.00	0.91	2.36	0.02
		post-test	3.05	0.82		
Evaluate	Experimental group	pre-test	3.03	0.97	0.10	0.92
		post-test	3.36	0.50		
	Control group	pre-test	3.01	0.97	0.01	0.02
		post-test	3.01	0.97		
Create	Experimental group	pre-test	2.54	0.84	− 0.06	0.95
		post-test	2.99(	0.63		
	Control group	pre-test	2.55	0.84	2.95	0.01
		post-test	2.63	0.62		

In the post-test phase, as delineated in Table 7, the differences between the experimental group and the control group in the domains of remembering, understanding, applying, analyzing, evaluating, and creating, were statistically significant (*p* > 0.05). This indicates that the performance levels of the experimental group in these cognitive domains during the post-test were notably superior to those of the control group. However, the differences between the experimental and control groups in remembering, understanding, and applying did not reach statistical significance (*p* > 0.05).

Table 8 illustrates a comparison between the pre-test and post-test scores for the experimental group across the cognitive domains of remembering, understanding, applying, analyzing, evaluating, and creating. A significant statistical difference was observed (*p* > 0.05), indicating that the levels in these domains post-test were considerably higher than their respective pre-test levels in the experimental group.

**Table 8:** Pre-test and post-test in experimental group and the control group.

Dimension	Group	Test	*M*	*SD*	*t*	*p*
Remember	Experimental group	pre-test	3.03	0.97	− 3.87	0.00
		post-test	3.21	0.87		
	Control group	pre-test	3.01	0.97	− 2.39	0.02
		post-test	3.17	0.72		
Understand	Experimental group	pre-test	3.06	0.96	− 2.76	0.01
		post-test	3.22	1.05		
	Control group	pre-test	3.06	0.97	− 2.19	0.03
		post-test	3.19	0.76		
Apply	Experimental group	pre-test	3.04	0.96	− 2.93	0.01
		post-test	3.21	1.05		
	Control group	pre-test	3.02	0.96	− 2.73	0.01
		post-test	3.20	0.64		
Analyze	Experimental group	pre-test	3.02	0.91	− 4.99	0.00
		post-test	3.38	0.61		
	Control group	pre-test	3.00	0.91	− 1.74	0.09
		post-test	3.05	0.82		
Evaluate	Experimental group	pre-test	3.03	0.97	− 3.50	0.01
		post-test	3.36	0.50		
	Control group	pre-test	3.01	0.97	− 0.53	0.59
		post-test	3.06	0.61		
Create	Experimental group	pre-test	2.54	0.84	− 4.09	0.00
		post-test	2.99	0.63		
	Control group	pre-test	2.55	0.84	− 1.08	0.28
		post-test	2.63	0.62		

In contrast, for the control group, Table 8 shows that the differences between pre-test and post-test scores in the domains of remembering, understanding, applying, analyzing, evaluating, and creating were not statistically significant (*p* > 0.05). However, significant statistical differences were found for the dimensions of remember, understand, and apply (*p* > 0.05). These results suggest that the post-test levels of these three dimensions in the control group were significantly higher than their respective pre-test levels.

The observation that the dimensions of remember, understand, and apply did not show statistically significant differences in the post-experiment test is consistent with the study by Smuts and Hattingh^68^. They posited that students exhibit more profound comprehension of critical thinking and higher-order cognition in the study of information skills courses, while the foundational results of lower-order thinking are not highlighted. Our experimental outcomes also align with the research by Hemalatha et al.^32^, which suggested that social media does not directly impact effective learning, but is significantly positively related to learning outcomes through mediating support and indirect effects.

Walle^69^ also asserted that when e-courses fail to meet students' needs, learning may be curtailed, leading to insignificant effects on students' basic learning outcomes. This phenomenon was further confirmed through student interviews, where the experimental group participants shared that BKSA appeared more effective in enhancing their higher-order learning outcomes and also improving lower-order learning outcomes within the traditional teacher-centered classroom context.

### The effects of BKSA on creativity

To assess the effectiveness of the Blended Knowledge Sharing Activities (BKSA), a mixed-methods approach was utilized, employing both questionnaires and interviews as tools for data collection. The questionnaire was designed to analyze the differences in creativity among students in the experimental and control groups post-intervention, as well as to compare the levels of creativity in the experimental group before and after the experiment.

In addition to the questionnaires, student interviews were conducted to gather their perceptions, feedback, and suggestions concerning the implementation of BKSA. This qualitative data further enabled us to refine and consider future practical applications of BKSA based on the students' experiences and perspectives.

As per the data shown in Table 9, during the pre-test phase, the experimental and control groups demonstrated no statistically significant differences (*p* < 0.05) in their divergent thinking, talent application abilities, and personality traits, implying that the two groups were homogeneous at the outset of the experiment.

**Table 9:** Pre-test in experimental and control group.

Dimension	Group dimension	*N*	*M*	*SD*	*t*	*p*
Divergent thinking	Experimental group	53	3.03	0.93	0.03	0.98
	Control group	52	3.02	0.94		
Ability to apply talents	Experimental group	53	3.07	0.94	0.01	0.99
	Control group	52	3.06	0.95		
Personality traits	Experimental group	53	3.01	0.98	− 0.18	0.86
	Control group	52	3.04	0.78		

Inspection of Table 10 reveals that for the experimental group, there were statistically significant differences (*p* < 0.05) in divergent thinking, talent application abilities, and personality traits between the pre-test and post-test results. Specifically, the post-test scores of the experimental group in divergent thinking, ability to apply talents, and personality traits were significantly elevated compared to their respective pre-test levels.

**Table 10:** Pre-test and post-test in the experimental group.

Dimension	Test	*M*	*SD*	*t*	*p*
Divergent thinking	Pre-test	3.03	0.93	− 5.64	0.00
	Post-test	3.40	0.68		
Ability to apply talents	Pre-test	3.07	0.94	− 3.83	0.00
	Post-test	3.40	0.5		
Personality traits	Pre-test	3.01	0.98	− 4.37	0.00
	Post-test	3.36	0.6		

Table 11 illustrates that for the control group, the differences in divergent thinking and talent application abilities between the pre-test and post-test were not statistically significant (*p* < 0.05). Turning to Table 12, it demonstrates that in the post-test, significant differences (*p* < 0.05) were observed between the experimental and control groups across divergent thinking, talent application abilities, and personality traits. The results indicate that the experimental group's levels of divergent thinking, talent application, and personality traits in the post-test were significantly higher than those in the control group.

**Table 11:** Pre-test and post-test in the control group.

Dimension	Test	*M*	*SD*	*t*	*p*
Divergent thinking	Pre-test	3.02	0.94	− 1.43	0.16
	Post-test	3.06	0.87		
Ability to apply talents	Pre-test	3.06	0.95	− 1.61	0.11
	Post-test	3.10	0.9		
Personality traits	Pre-test	3.04	0.78	− 1.17	0.25
	Post-test	3.08	0.74

**Table 12:** Post-test in experimental and control group.

Dimension	Group	*N*	*M*	*SD*	*t*	*p*
Divergent thinking	Experimental group	53	3.40	0.68	2.21	0.03
	Control group	52	3.06	0.87		
Ability to apply talents	Experimental group	53	3.40	0.50	2.12	0.04
	Control group	52	3.10	0.90		
Personality traits	Experimental group	53	3.36	0.60	2.121	0.04
	Control group	52	3.08	0.74		

### Analysis of structural equation model

The choice of data analysis methods varies among researchers: some utilize T-tests^70^, while others employ Structural Equation Modeling (SEM)^70^. This study initially employed both T-tests and SEM. We found that the predictions generated by these two analytical methods generally align. Moreover, a succinct analysis of the structural equations has been incorporated. This research employed confirmatory factor analysis to verify the construct validity of pertinent variables, which include four processes in the SECI model, six variables in Bloom's taxonomy, and three creativity variables. The Structural Equation Modeling (SEM) was used to analyze the impact of the SECI process and knowledge sharing on learning outcomes and creativity. The results of this analysis are depicted in the following figure.

**Figure 4:**
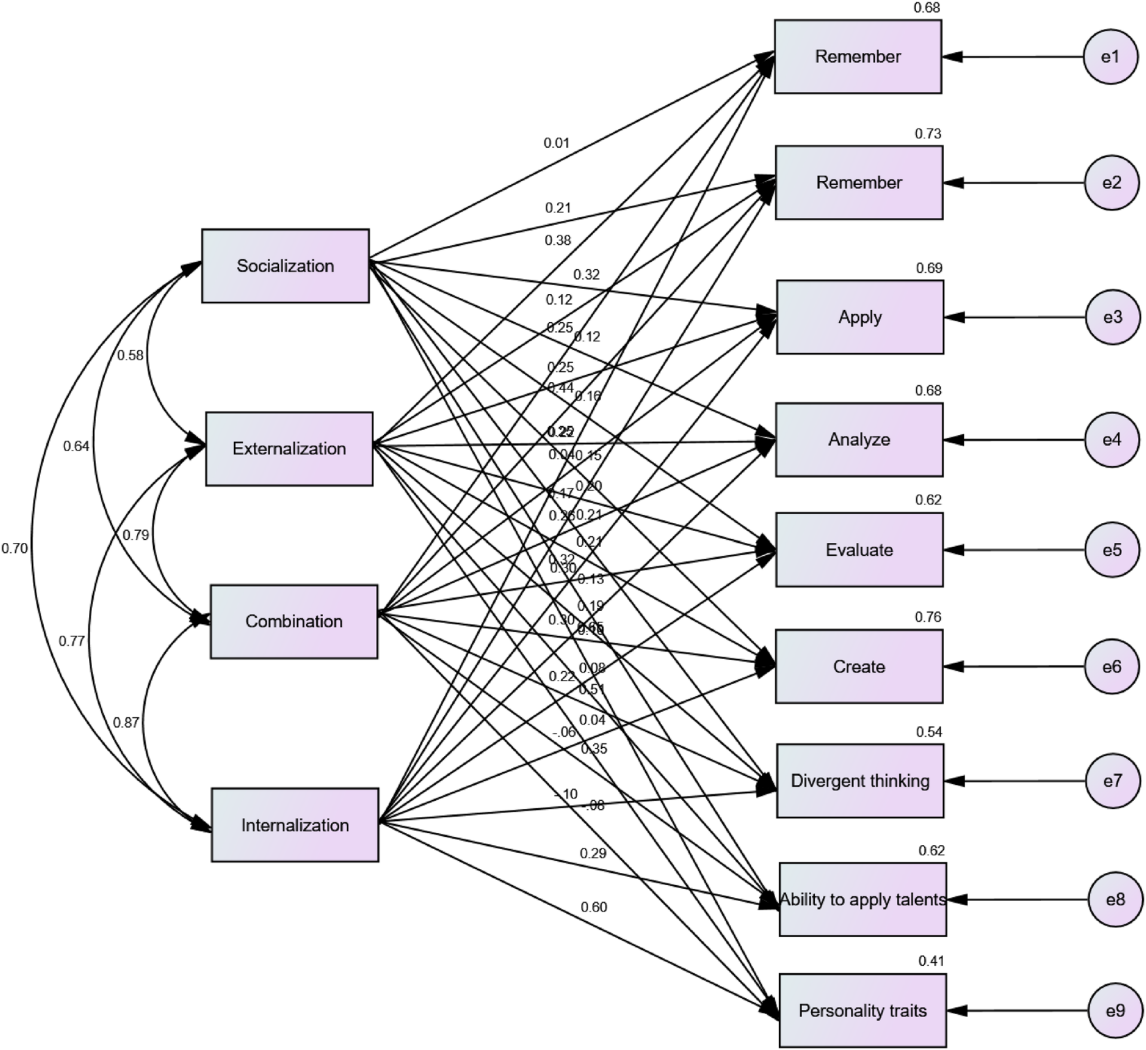
Structured analysis methods of BKSA.

Table 13 and Fig. 4 depict that socialization positively influences understanding, application, analysis, evaluation, creation, divergent thinking, and the ability to apply intelligence (*p* < 0.05). Externalization has a significantly positive impact on memory, comprehension, application, analysis, evaluation, creation, and divergent thinking (*p* < 0.05) Moreover, combination significantly enhances memory, comprehension, analysis, evaluation, creation, divergent thinking, and the capacity to apply intellect (*p* < 0.05). Lastly, internalization has a marked positive effect on memory, comprehension, application, analysis, evaluation, ability to apply intelligence, and personality traits (*p* < 0.05).

**Table 13:** Path analysis results.

Path	Estimate	S.E	C.R	P
Socialization → Remember	0.007	0.053	0.142	0.887
Socialization → Understand	0.208	0.044	4.613	***
Socialization → Apply	0.324	0.042	6.718	***
Socialization → Analyze	0.120	0.043	2.459	0.014
Socialization → Evaluate	0.159	0.047	2.966	0.003
Socialization → Create	0.152	0.038	3.609	***
Socialization → Divergent thinking	0.210	0.053	3.593	***
Socialization → Ability to apply talents	0.134	0.05	2.508	0.012
Socialization → Personality traits	0.100	0.069	1.506	0.132
Externalization Remember	0.380	0.062	6.505	***
Externalization → Understand	0.122	0.052	2.261	0.024
Externalization → Apply	0.249	0.049	4.298	***
Externalization → Analyze	0.223	0.05	3.790	***
Externalization → Evaluate	0.199	0.055	3.106	0.002
Externalization → Create	0.208	0.045	4.123	***
Externalization → Divergent thinking	0.195	0.062	2.784	0.005
Externalization → Ability to apply talents	0.083	0.059	1.286	0.199
Externalization → Personality traits	0.044	0.081	0.547	0.585
Combination → Remember	0.246	0.082	3.204	0.001
Combination → Understand	0.443	0.069	6.23	***
Combination → Apply	0.041	0.065	0.536	0.592
Combination → Analyze	0.261	0.066	3.376	***
Combination → Evaluate	0.297	0.072	3.513	***
Combination → Create	0.645	0.059	9.719	***
Combination → Divergent thinking	0.511	0.081	5.553	***
Combination → Ability to apply talents	0.352	0.078	4.166	***
Combination → Personality traits	− 0.076	0.106	− 0.722	0.47
Internalization → Remember	0.254	0.087	3.228	0.001
Internalization → Understand	0.169	0.073	2.325	0.02
Internalization → Apply	0.321	0.069	4.127	***
Internalization → Analyze	0.303	0.071	3.835	***
Internalization → Evaluate	0.217	0.077	2.509	0.012
Internalization → Create	− 0.058	0.063	− 0.86	0.39
Internalization → Divergent thinking	− 0.102	0.087	− 1.078	0.281
Internalization → Ability to apply talents	0.286	0.083	3.306	***
Internalization → Personality traits	0.597	0.113	5.557	***

### Analysis of interviews

Results and analysis of student interviews. At the end of the experimental study, ten students from the experimental group, which were randomly selected from the experimental group on a voluntary basis, are interviewed via WeChat. The interviews focused on three questions and the results can be divided into three parts: (i) the advantages of BKSA; (ii) what the students gained from BKSA; and (iii) suggestions for BKSA. Conversations are transcribed, and data are coded and analyzed. After the interviews, participating students found the BKSA format to be highly interactive. The collaboration of the group, the positive interaction with the network, and the recognition of the students and the network made the students more interested in the expertise. After using BKSA, teaching resources become rich in teaching in learning. BKSA can enhance self-learning ability, solidify professional skills, increase the value of interaction and enhance creativity. For the suggestions of BKSA, the participating students think they should stipulate the learning content and create works in combination with other courses.

**Figure 5:**
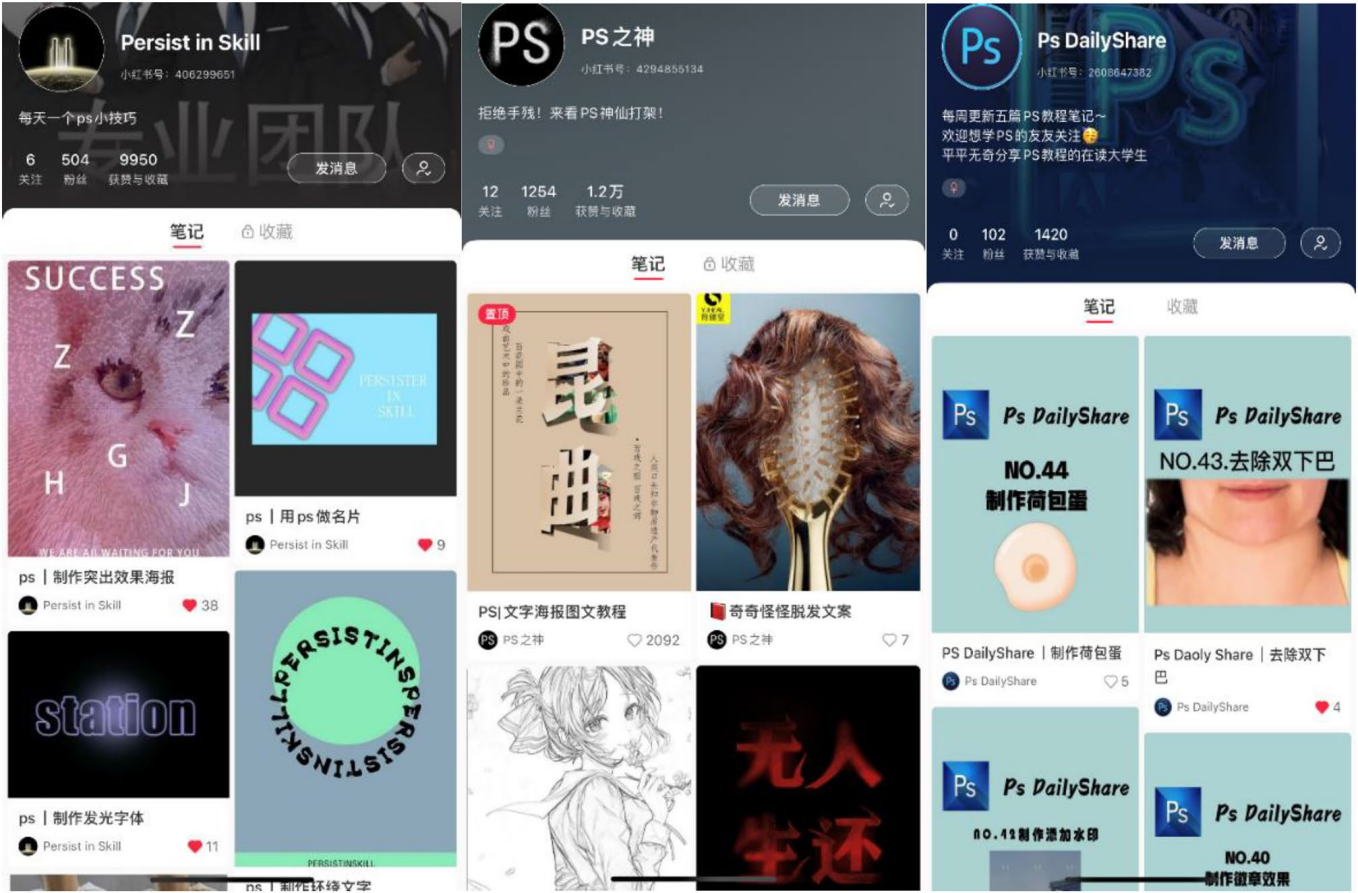
Students’ knowledge sharing results on social media (partial excerpts).

The measurement of this study does not only rely on own learning outcomes, but also includes the homework of students in the experimental group. The homework is in the form of each group's study sharing account on social media. Students share their learning results on social media and interact with netizens. Netizens’ positive comments, likes, and attention to knowledge sharing content are all the experimental results of this BKSA. Figure 5 illustrates that BKSA had a positive impact on learning outcomes and creativity.

## Conclusion

This paper presents an enhanced Blended Knowledge Sharing Activity (BKSA) model, predicated on Nonaka and Takeuchi^51,52^ Theory of Knowledge Transformation and Creation, incorporating both online and offline Ba elements. Building on the blended learning activity model, a BKSA study was undertaken in the context of a design program. Utilizing extant studies and expert interviews, the BKSA activities were structured into eleven activity strategies grounded in the four dimensions of socialization, externalization, combination, and internalization, as proposed in the SECI model. The impact of these blended knowledge sharing activities on learning outcomes and creativity was explored and analyzed through their implementation among design students.

A measurement scale for BKSA, drawing on the SECI model^58^, was devised to facilitate the analysis of theoretical and practical aspects of knowledge sharing across multi-source datasets. Learning outcomes were assessed using scales designed on the basis of Bloom et al.^71^ analysis of cognitive learning outcomes and Pickard^66^ framework. The creativity questionnaire was divided into three dimensions: divergent thinking, ability to apply talents, and personality traits^67^. Furthermore, adopting the research methodology of Yilmaz and Karaoglan Yilmaz^19^, the data obtained from the BKSA, Learning Effectiveness, and Creativity Questionnaire were analyzed employing IBM SPSS Statistics software.

This study scrutinizes the effectiveness of Blended Knowledge Sharing Activity (BKSA) from both quantitative and qualitative perspectives. Quantitatively, post-BKSA application, the experimental group displayed marked improvements in learning outcomes and creative abilities of design students, outperforming the control group significantly. This illustrates the enhancement in BKSA-related behaviors leading to improved learning outcomes and creativity among design students. Qualitatively, the students and teachers expressed general satisfaction with the BKSA implementation, attributing it to enhanced self-learning, amplified interactional value, and improved creativity. Furthermore, the interviews facilitated in strengthening teacher-student relationships and encouraged suggestions for developing sharing platforms. The BKSA variables' effects on creativity were empirically examined to discern the SECI model's role as a direct (main effect) or indirect (mediating effect), thereby contributing to our comprehension of how the SECI model influences creativity.

Educators and researchers are inclined to adopt novel instructional models that meet current pedagogical expectations, encompassing social media, communities, flipped classrooms, virtual reality, among others^18–20,59,60,72^. The enhancement of knowledge sharing behaviors among students can elevate knowledge creation and creativity at both team and individual levels. By fully immersing in the creative process and devoting substantial attention to a problem, individuals may yield creative solutions^49^. By discerning problems from various perspectives and amassing diverse yet relevant information, students can create manifold options. A trusting environment facilitates wide-ranging information and knowledge sharing^73^. Yilmaz and Karaoglan Yilmaz^19^ offers valuable insights into blended knowledge sharing activities, underscoring the benefits and challenges of blended learning and strategies for effectively designing and implementing blended learning environments. Furthermore, the impacts of metacognitive awareness, reflective thinking, problem-solving, and communities of inquiry on students' self-efficacy, and the interrelationships among them, provide crucial guidance for understanding how students can augment their self-efficacy, technological competency, and advanced thinking skills within blended learning contexts^18^.

While our study yields positive findings, it is not devoid of limitations. Firstly, the methodologies employed within this study predominantly consist of questionnaires and interviews, which may be subjected to students' subjectivity. Future research might consider the use of more empirical tools and methodologies, such as the tracking of students' online activity data, to achieve a more in-depth and comprehensive comprehension.

Secondly, the statistical results, being sample-based, may not provide an entirely representative view of all design undergraduates. Furthermore, the selection of our study sample from students enrolled in specific courses might have introduced a selection bias.

Despite these constraints, our study furnishes novel perspectives for comprehending the impact of blended knowledge-sharing activities on design undergraduates. Our findings suggest that blended knowledge-sharing activities can function as an efficacious educational strategy to enhance student learning outcomes and creativity. Future research should further explore this domain, thereby deepening our understanding and advancing pedagogical practices.

## Data Availability

The data that support the findings of this study are available from the corresponding authors upon reasonable request.
